# A novel thermostable chitinolytic machinery of *Streptomyces* sp. F-3 consisting of chitinases with different action modes

**DOI:** 10.1186/s13068-019-1472-1

**Published:** 2019-06-03

**Authors:** Xiaomeng Sun, Yingjie Li, Zhennan Tian, Yuanchao Qian, Huaiqiang Zhang, Lushan Wang

**Affiliations:** State Key Laboratory of Microbial Technology, Microbial Technology Institute, Shandong University, No. 72 Jimo Binhai Road, Qingdao, 266237 Shandong People’s Republic of China

**Keywords:** *Streptomyces* sp. F-3, Thermophilic chitinase, GH18 sub-family, Action modes, Synergy

## Abstract

**Background:**

The biodegradation of chitin is an important part of the carbon and nitrogen cycles in nature. Speeding up the biotransformation of chitin substrates can not only reduce pollution, but also produce high value-added products. However, this process is strictly regulated by the catalytic efficiency of the chitinolytic machinery. Therefore, it is necessary to study the mode of action and compound mechanisms of different chitin-degrading enzymes in depth to improve the catalytic efficiency of the chitinolytic machinery.

**Results:**

The thermophilic bacterium *Streptomyces* sp. F-3 showed comparatively high chitin degradation activities. To elucidate the mechanism underlying chitin hydrolysis, six chitin degradation-related enzymes were identified in the extracellular proteome of *Streptomyces* sp. F-3, including three chitinases (*Ss*Chi18A, *Ss*Chi18B, and *Ss*Chi18C) from the GH18 family, one GH19 chitinase (*Ss*Chi19A), one GH20 β-*N*-acetylhexosaminidase (*Ss*GH20A), and one lytic polysaccharide monooxygenase (*Ss*LPMO10A) from the AA10 family. All were upregulated by chitin. The heterologously expressed hydrolases could withstand temperatures up to 70 °C and were stable at pH values of 4 to 11. Biochemical analyses displayed that these chitin degradation-related enzymes had different functions and thus showed synergistic effects during chitin degradation. Furthermore, based on structural bioinformatics data, we speculated that the different action modes among the three GH18 chitinases may be caused by loop differences in their active site architectures. Among them, *Ss*Chi18A is probably processive and mainly acts on polysaccharides, while *Ss*Chi18B and *Ss*Chi18C are likely endo-non-processive and displayed higher activity on the degradation of chitin oligosaccharides. In addition, proteomic data and synergy experiments also indicated the importance of *Ss*LPMO10A, which could promote the activities of the hydrolases and increase the monosaccharide content in the reaction system, respectively.

**Conclusions:**

In this article, the chitinolytic machinery of a thermophilic *Streptomyces* species was studied to explore the structural basis for the synergistic actions of chitinases from different GH18 subfamilies. The elucidation of the degradation mechanisms of these thermophilic chitinases will lay a theoretical foundation for the efficient industrialized transformation of natural chitin.

**Electronic supplementary material:**

The online version of this article (10.1186/s13068-019-1472-1) contains supplementary material, which is available to authorized users.

## Introduction

Chitin, a polymer of β-1,4-*N*-acetylglucosamine, is the second most abundant polysaccharide in nature after cellulose [1]. It is mainly derived from fungal cell walls, insect exoskeletons, and crustaceans. An estimated 10^11^ tons of chitin are produced in water per year [2]. Chitin can be biotransformed into pharmacologically active products, such as *N*-acetylglucosamine and chitin oligosaccharides (CHOSs), which can be used as antimicrobial agents and immune enhancers to activate the host defense system. These substances can also be used as drug delivery vehicles and antioxidants, which could be useful in hemostasis, wound healing, blood cholesterol control, and food preservation [3], as well as have important anti-tumor and anti-infection activities [4]. Therefore, as an abundant natural biomass resource, the degradation and transformation of chitin have become hot research topics.

Chitinases are often used to degrade chitin in crustaceans and fungal cell walls, including chitin hydrolases and lytic polysaccharide monooxygenases (LPMOs). On the basis of hydrolytic characteristics, chitin hydrolases can be classified into three types: *endo*-chitinase (EC 3.2.1.14), *exo*-chitinase (EC 3.2.1.29), and β-*N*-acetylglucosaminidase (EC 3.2.1.52) [5]. Some chitinases act processively on single chains stripped from crystal substrates and thus play an important role in the degradation of insoluble substrates [6]. The LPMO is capable of catalyzing the oxidative cleavage of glucosidic bonds, thereby altering the supramolecular structures and increasing the accessibility and degradability of substrates [7].

In the CAZy database (http://www.cazy.org/), most chitin hydrolases belong to glycoside hydrolase (GH) families 18, 19, and 20. The chitinases from different GH families have unique three-dimensional structures and substrate-binding patterns [8, 9]. In addition, chitinases, especially bacterial chitinases, usually have multiple auxiliary functional domains, including a carbohydrate-binding module (CBM), which enhances the binding abilities of enzymes to insoluble substrates, and a fibronectin type III (FN3) domain, which increases the stability of enzymes [10].

The degradation of recalcitrant crystalline chitin requires the participation of multiple enzymes. One representative enzyme system is produced by *Serratia marcescens* [11]. The end of the chain produced by *endo*-chitinases could provide a new binding site for *exo*-chitinases, thereby increasing the efficiency of enzyme degradation. Recent reports noted that LPMOs in both *S. marcescens* and *Cellvibrio japonicus* are able to significantly increase the efficiency of chitin hydrolases for the degradation of crystalline chitin and thus initiate the degradation of natural chitin [7, 12].

In industrial processes, thermostable enzymes have been the focus of research because of their wide applications and low costs [2, 13]. However, so far, the chitinases used in industrial applications are mainly derived from the mesophilic bacteria *S. marcescens* and *C. japonicus* [14, 15]. The optimum temperatures of the extracellular chitinases of these Gram-negative bacteria are 50 °C [16] and 30 °C [15], respectively. The optimum pH is 6.5, which is close to neutral [12, 17]. Therefore, screening and studying the properties and compositions of thermophilic enzymes, and elucidating the functions and synergies of various components of the enzyme system have become important topics of related research.

Recently, we isolated a thermophilic bacterium *Streptomyces* sp. F-3 from soil which can grow rapidly at 50 °C [18]. When colloidal chitin was used as the sole carbon substrate, increased chitinase activity was observed. Here, we set out to investigate the mechanism of chitin degradation. First, we heteroexpressed all chitin-degrading enzymes, which were secreted extracellularly. Then, their biochemical characteristics and functions were examined to explore the mechanism of chitin degradation for prospective industrial applications. Moreover, bioinformatic analysis of the GH18 chitinases was carried out to distinguish their degradation mechanisms. Overall, our work aims at studying a novel thermostable chitinolytic machinery in order to obtain a new thermostable enzyme system for industrial applications.

## Results

### Identification of enzymes involved in chitin degradation

**Fig. 1:**
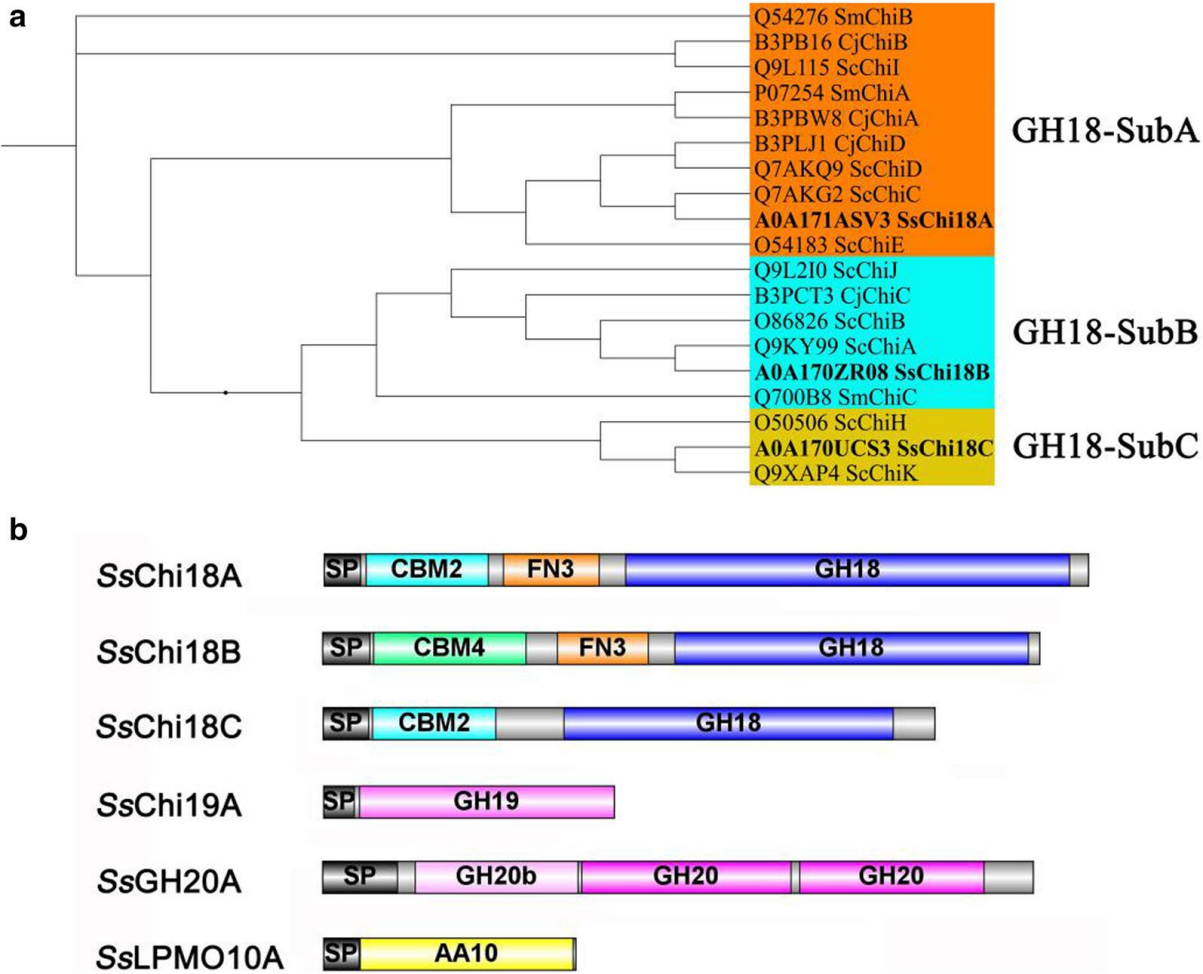
Sequence analysis of the chitin degradation-related enzymes from *Streptomyces* sp. F-3. **a** Phylogenetic tree analysis of *Ss*Chi18A, *Ss*Chi18B, and *Ss*Chi18C. The putative catalytic domain was compared with those of *S. coelicolo*r, *C. japonicus*, and *S. marcescens*. **b** The architecture of the secreted chitin degradation-related proteins of *Streptomyces* sp. F-3. The indicated domains are shown as follows: GH18, GH19, GH20, AA10, catalytic domains; CBM2 and CBM4, chitin-binding domain; FN3, fibronectin domain; SP, signal peptide. There are three domains in *Ss*Chi18A, including CBM2, FN3, and GH18 from the N-terminus to the C-terminus, respectively. *Ss*Chi18B contains CBM4, FN3, and GH18; *Ss*Chi18C contains CBM2 and GH18. *Ss*Chi19A and *Ss*LPMO10A have only one catalytic domain from the GH19 and AA10 family, respectively. For *Ss*GH20A, there are three domains, a bacterial type N-terminal β-hexosaminidase (GH20b) and two catalytic domains from glycoside hydrolase family 20

Since *Streptomyces* sp. F-3 is able to withstand high temperatures, at which comparatively high activity of chitin degradation was observed, genes coding for chitin metabolism were analyzed. Nine chitin degradation-related enzymes were identified in the genome of *Streptomyces* sp. F-3, including three chitinases from the GH18 family, one from the GH19 family, three β-*N*-acetylhexosaminidases from the GH20 family, and two LPMOs from the AA10 family, of which, seven were predicted as extracellular (Additional file 1: Table S1). Although three hydrolases belong to the GH18 family, they can be classified into three subfamilies based on their catalytic domains [19]: *Ss*Chi18A belonging to the sub-family A, *Ss*Chi18B from the sub-family B, and *Ss*Chi18C from the sub-family C (Fig. 1a).

To confirm the bioinformatics data, we investigated the extracellular chitin-degrading compositions of *Streptomyces* sp. F-3 by mass spectrometry. Different from the bioinformatics data, only six related chitin-degrading proteins were observed, while one AA10 LPMO (*Ss*LPMO10B) was absent (Additional file 1: Table S1). Among these GH18 chitinases, *Ss*Chi18A showed the highest expression level and accounted for up to 2.1% of extracellular proteins, followed by *Ss*Chi18B (0.82%) and *Ss*Chi18C (0.01%). The GH19 family *Ss*Chi19A accounted for 0.4% of all extracellular proteins. In addition, the putative β-*N*-acetylhexosaminidase *Ss*GH20A accounted for 1.17% and the LPMO designated *Ss*LPMO10A for about 0.65% of the extracellular proteins (Additional file 1: Table S1). Altogether, our data showed that six extracellular chitin-degrading proteins identified by proteome, displayed different expression levels in vivo and harbor distinct auxiliary and catalytic domains (Fig. 1b), suggesting they may play different roles in the chitin degradation process.

### Characterization of extracellular chitinases in vitro

Because the secreted chitin degradation-related proteins had different architectures and expression levels based on bioinformatics and proteomic analysis, heterologous expression and purification were conducted to distinguish the function of the six enzymes. First, the relative molecular weights of related proteins were examined by sodium dodecyl sulfate electrophoresis (Additional file 1: Fig. S1). Since *Ss*LPMO10A showed no chitin-hydrolyzing activity and no reducing end was generated (data not shown), the optimum temperature and pH were not determined.

**Fig. 2:**
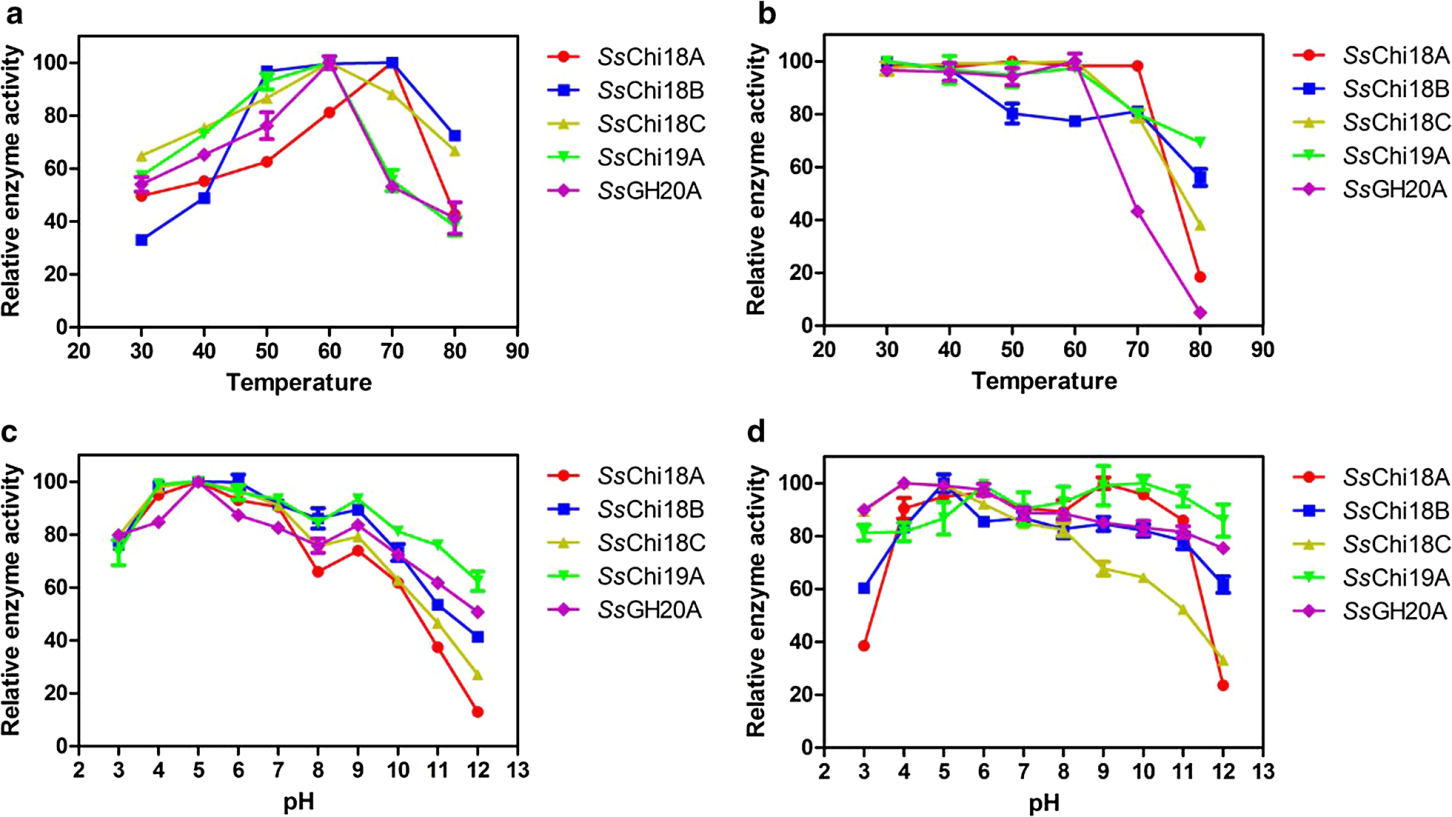
Effect of temperature and pH on chitinases of *Streptomyces* sp. F-3. **a** The optimum reaction temperature of the chitinases. Chitinase activity was measured with colloidal chitin at temperatures ranging from 30 to 80 °C at pH 5.0 for 30 min. **b** The temperature stability of the chitinases. The temperature stability was determined by measuring the remaining activity after incubation at various temperatures at pH 5.0 for 30 min without substrate. **c** The optimum reaction pH. Chitinase activity was determined at various pH values (pH 3–8, 50 mM Na_2_HPO_4_-citric acid buffer; pH 9–12, 50 mM Na_2_HPO_4_–NaOH buffer) at 60 °C for 30 min. **d** The pH stability of the chitinases. The pH stability was determined by measuring the residual activity after 30 min incubation at various pH values at 20 °C without substrate. Error bars are given as means and standard deviations

The optimum temperature was 70 °C for the enzymatic activities of *Ss*Chi18A and *Ss*Chi18B, and was 60 °C for *Ss*Chi18C, *Ss*Chi19A, and *Ss*GH20A (Fig. 2a). These results suggested that these enzymes are thermophilic. However, they had different tolerances to high temperatures. As shown in Fig. 2b, although *Ss*Chi18A showed the highest thermostability and could withstand temperatures of up to 70 °C, its activity was sharply decreased to less than 20% at temperatures above 80 °C, possibly due to heat inactivation or protein degradation. *Ss*Chi18C, *Ss*Chi19A, and *Ss*GH20A could withstand a temperature of 60 °C, and only retained 40%, 70%, and 5% of their respective activities after incubation at 80 °C for 30 min (Fig. 2b). *Ss*Chi18B was stable at 30–40 °C, which was less thermostable than the other four chitinases, and retained 80% of its activity at 50–70 °C (Fig. 2b).

The optimal pH of these chitinases was 5 (Fig. 2c), but the enzymes retained 85% of their activities at pH 4–6. Among them, *Ss*Chi19A and *Ss*GH20A had the best pH tolerance, and were stable at pH 3–12 after incubation for 30 min without substrate (Fig. 2d). *Ss*Chi18A and *Ss*Chi18B were relatively stable at pH 4–11, but their stabilities had decreased in extreme acidic conditions (pH 3) and in the presence of a polar base (pH 12). *Ss*Chi18C retained more than 80% activity at pH 3–8 and showed lower tolerance to more alkaline conditions (pH 9–12). Therefore, our data indicated that the secreted chitinases in *Streptomyces* sp. F-3 were not only thermophilic, but also displayed a broader tolerance to pH changes compared to the fungal chitinases (stable at pH 4–8, [20]), suggesting their potential use in industrial applications.

### Chitinases have different activities toward chitin polymers and CHOSs

To further investigate the features of the chitinases, substrate specificity was measured. All five chitinases were able to hydrolyze colloidal chitin and powdered chitin, but not chitosan with a low degree of acetylation (Table 1). Among them, *Ss*Chi18A showed the highest activity. Compared to their activities on soluble colloidal chitin, the enzymatic activities on insoluble chitin powder were much lower. These data indicate that the efficiency of the chitinases is limited by the accessibility of the substrate, as reported by Andersen et al. [21].

**Table 1:** Substrate specificity of chitinases from *Streptomyces* sp. F-3

Substrate	Specific activity (U/μmol)^a^				
	*Ss*Chi18A	*Ss*Chi18B	*Ss*Chi18C	*Ss*Chi19A	*Ss*GH20A
Colloidal chitin	54.33 ± 0.55	34.74 ± 2.72	28.61 ± 2.16	5.09 ± 0.83	1.97 ± 0.18
Chitin powder	4.81 ± 0.14	1.75 ± 0.10	1.50 ± 0.15	2.02 ± 0.26	0.97 ± 0.57
Chitosan^b^	0.41 ± 0.06	0.19 ± 0.08	0.28 ± 0.02	0.22 ± 0.07	0.09 ± 0.07
CMC	ND	ND	ND	0.090 ± 0.05	ND
(GlcNAc)6	3.9 ± 0.2 × 10^3^	10.8 ± 0.5 × 10^3^	11.6 ± 0.8 × 10^3^	6.9 ± 0.6 × 10^3^	89.4 ± 1.0 × 10^3^
(GlcNAc)5	4.6 ± 0.1 × 10^3^	7.7 ± 0.2 × 10^3^	8.9 ± 0.6 × 10^3^	6.5 ± 0.5 × 10^3^	63.8 ± 0.9 × 10^3^
(GlcNAc)4	4.8 ± 0.2 × 10^3^	7.0 ± 0.1 × 10^3^	15.1 ± 0.5 × 10^3^	9.9 ± 0.8 × 10^3^	93.6 ± 1.6 × 10^3^
(GlcNAc)3	6.3 ± 0.2 × 10^3^	8.0 ± 0.3 × 10^3^	5.3 ± 0.2 × 10^3^	4.0 ± 0.3 × 10^3^	76.3 ± 1.0 × 10^3^
(GlcNAc)2	ND	ND	ND	ND	68.7 ± 0.6 × 10^3^

ND, not detectable

^a^Specific activity was obtained by detecting the generation of the reducing sugar after the reaction mixtures were incubated at 60 °C for 30 min. In the reaction system, the concentration of the polysaccharides was 10 mg/ml, and the concentration of the oligosaccharide substrates was 1 mg/ml. Results are presented as the mean ± standard error (*n* = 3)

^b^Chitosan, with a degree of acetylation of less than 10%

In addition, we also tested their chitinolytic activity toward CHOSs with degrees of polymerization ranging from 2 to 6 GlcNAc units (G2–G6). As shown in Table 1, *Ss*GH20A had the highest oligosaccharide-degrading activity, with an order of magnitude higher than other chitin hydrolases. Notably, except for *Ss*GH20A, none of the chitinases were able to degrade G2, suggesting that this dimer may be the final product of chitin hydrolysis while *Ss*GH20A may function as an *N*-acetylhexosaminidase.

### Different chitinases have different action modes toward chitin polymers and CHOSs

**Fig. 3:**
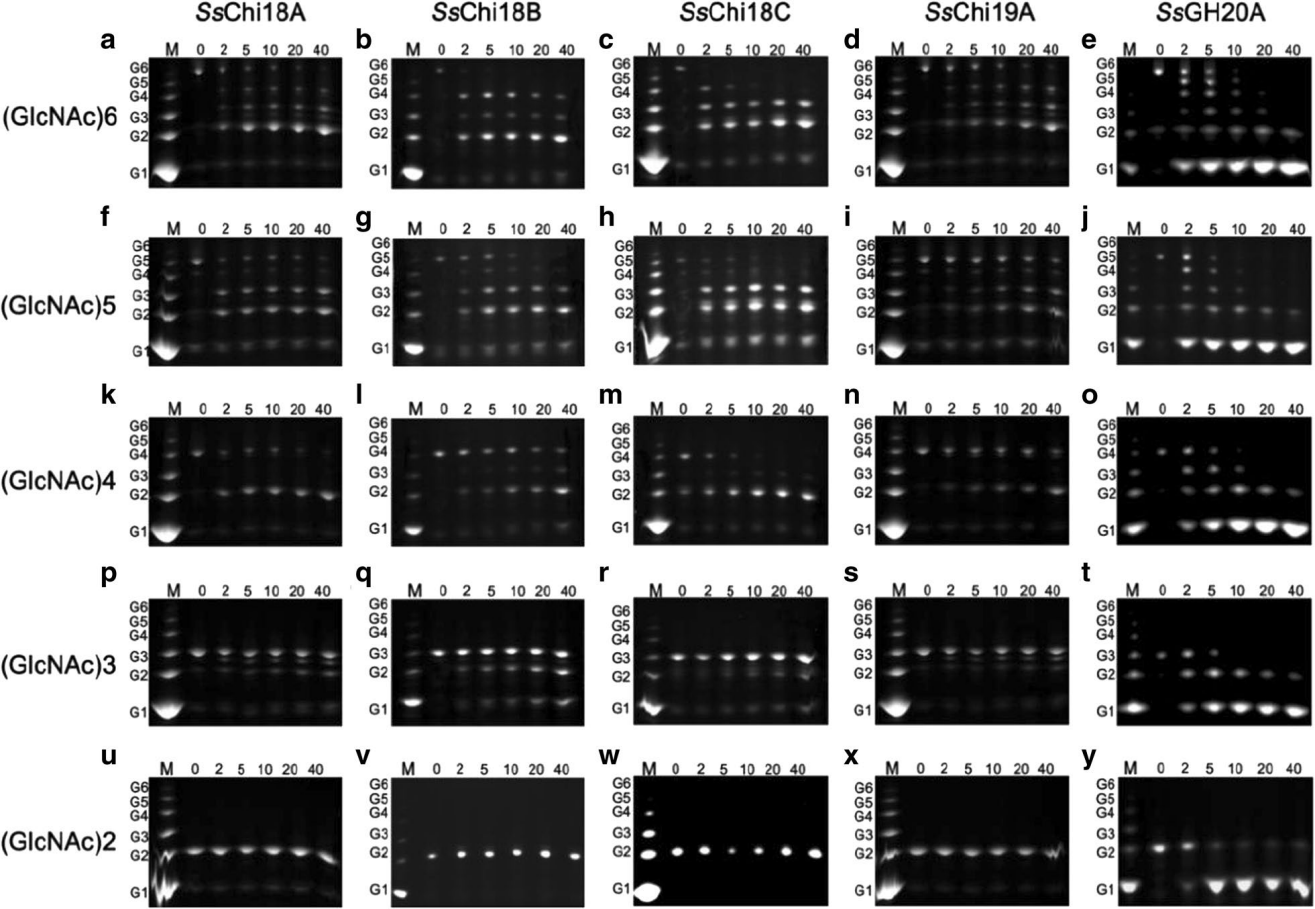
FACE analysis of hydrolysis products by chitinases from various CHOSs. The reaction mixture (100 μL) containing 60 μL of 1 mg/mL substrate was incubated with purified enzyme (40 μL, 0.01 nmol/mL) at 60 °C. The reaction mixture was analyzed at the indicated times. Standard CHOSs from GlcNAc (G1) to GlcNAc6 (G6). **a**, **f**, **k**, **p**, **u** For *Ss*Chi18A; **b**, **g**, **l**, **q**, **v** for *Ss*Chi18B; **c**, **h**, **m**, **r**, **w** for *Ss*Chi18C; **d**, **i**, **n**, **s**, **x** for *Ss*Chi19A; and **e**, **j**, **o**, **t**, **y** for *Ss*GH20A

Since different chitin-hydrolyzing activities were observed among the five enzymes, fluorescence-assisted carbohydrate electrophoresis (FACE) was performed to monitor the product types and contents of different substrates during degradation. The product profiles for *Ss*Chi18A and *Ss*Chi19A toward colloidal chitin were mainly G2 and G1, with visible amounts of G3 (Additional file 1: Fig. S2), a common feature for GH18 chitinases [22, 23]. In the product profiles of *Ss*Chi18B and *Ss*Chi18C, besides the major products G2 and G1, G4 and G5 were also accumulated, indicating that the functions of *Ss*Chi18B and *Ss*Chi18C may be different from that of *Ss*Chi18A. Therefore, to understand the roles of *Ss*Chi18B and *Ss*Chi18C during chitin degradation, the product profiles toward soluble CHOSs (G2–G6) were also tested to give insight into the preferred substrate-binding modes. As shown in Fig. 3a, the G6 hydrolysis products yielded by *Ss*Chi18A were G2, G3, and G4, with G2 being the major one (Fig. 3a). However, the G6 degradation products of *Ss*Chi18B were firstly G2, G3, and G4, and subsequently, partial G4 was hydrolyzed to G2 (Fig. 3b). Similar but greater catalytic activities were observed for *Ss*Chi18C, suggesting a non-processive reaction (Fig. 3c). When G4 was used as a substrate, *Ss*Chi18A only produced G2, whereas the product profiles of *Ss*Chi18B and *Ss*Chi18C included G2 and small amounts of G3 (Fig. 3k–m). When mixing with G3, only *Ss*Chi18B showed a significant effect. Although the hydrolysis modes of *Ss*Chi19A were similar with those of *Ss*Chi18A, its catalytic velocity was slower over time. *Ss*GH20A displayed a typical action manner of glycosidase, and stepwise hydrolyzed oligosaccharides to monosaccharides. Taken together, our observation revealed that although *Ss*Chi18A, *Ss*Chi18B, and *Ss*Chi18C belong to the GH18 family, the properties of *Ss*Chi18A differ from those of *Ss*Chi18B and *Ss*Chi18C.

### Chitinases from different GH18 subfamilies displayed different binding and catalytic modes

**Fig. 4:**
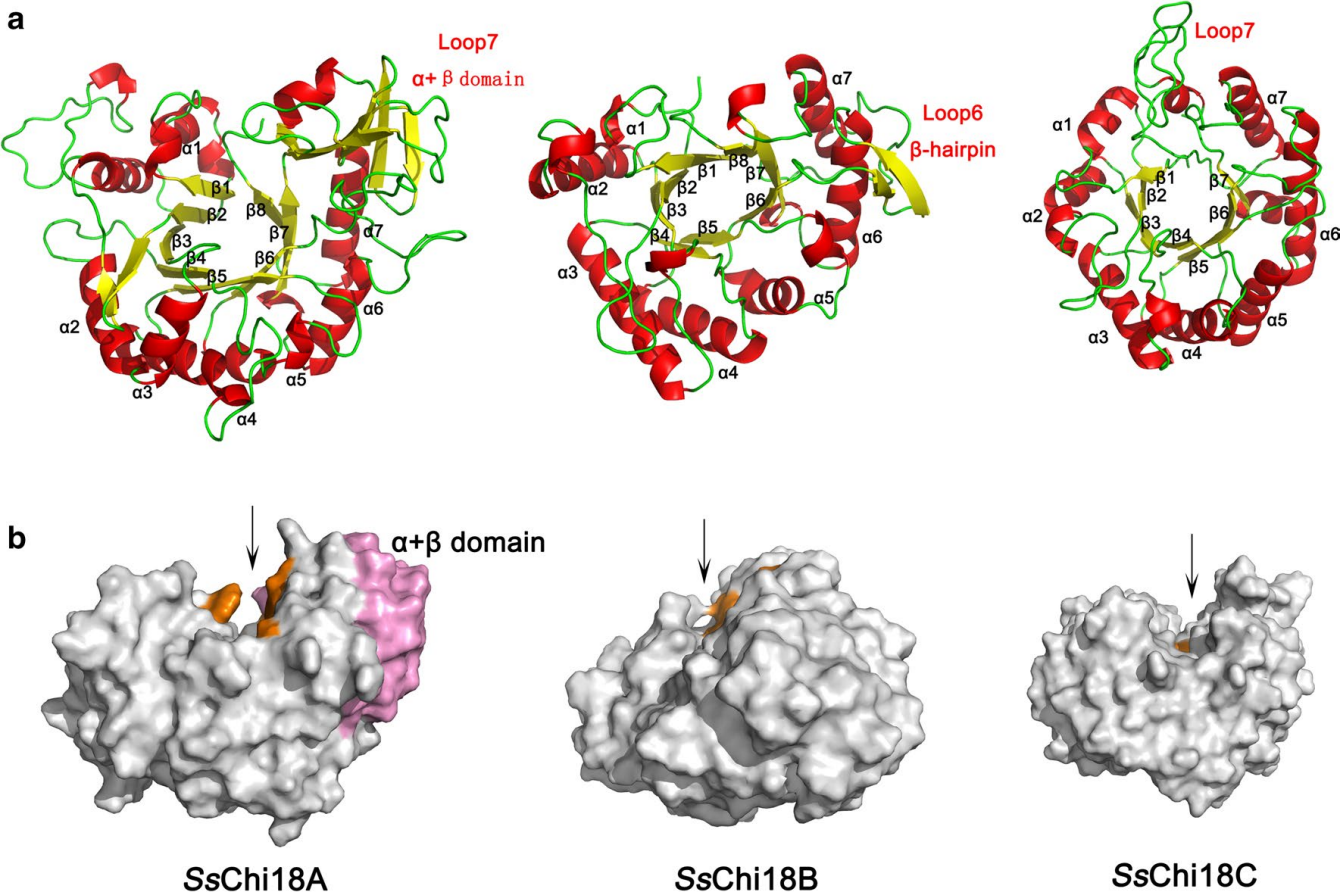
Structural bioinformatics analysis of the chitinases of *Streptomyces* sp. F-3. **a** Structural models of the catalytic domains of *Ss*Chi18A, *Ss*Chi18B, and *Ss*Chi18C. β-Sheets are shown in yellow, α-helices are shown in red and loops are shown in green. **b** The structural characteristics of the catalytic domains of *Ss*Chi18A, *Ss*Chi18B, and *Ss*Chi18C. Aromatic residues in the substrate-binding cleft are shown in orange, and the α + β domain is shown in pink

Since different hydrolyzing actions were observed among the GH18 chitinases, the PyMOL molecular visualization system (https://pymol.org/) was used to build models of the catalytic domains of *Ss*Chi18A, *Ss*Chi18B, and *Ss*Chi18C, to provide insight into the structural basis of these functional differences. We found that all three enzymes contain the typical GH18 structure of a TIM barrel, while some differences are present (Fig. 4). For example, *Ss*Chi18A and *Ss*Chi18B contain eight β-sheets in the active architectures, whereas *Ss*Chi18C contains only seven (Fig. 4a). Except for Phe-396 in the processive chitinase *Sm*ChiA from *S. marcescens*, which is Trp-437 and Trp-213 in the *Cj*Chi18D from *C. japonicus* and *Ss*Chi18A from *Streptomyces* sp. F-3, respectively [12, 24], the active center of *Ss*Chi18A contains the same aromatic amino acids with these two reported processive chitinases (Additional file 1: Fig. S3A), suggesting that *Ss*Chi18A is processive [24, 25]. Consistent with this, we also observed a conserved motif of SXGGW in the catalytic domain of *Ss*Chi18A (Additional file 1: Fig. S4), where the Trp has been demonstrated essential for processivity [26, 27]. In the active site architecture, a large difference was observed in the loop regions. For *Ss*Chi18A, there is a clear α + β subdomain insertion on loop 7, while *Ss*Chi18B has a β-hairpin on loop 6. Compared to *Ss*Chi18A and *Ss*Chi18B, although the eighth β-sheet is absent in *Ss*Chi18C, it contains a longer chain of loop 7, which occupies the position where the β8 sheet of *Ss*Chi18A and *Ss*Chi18B is located. Moreover, Trp-257 and Arg-261 in the loop 7 are possibly involved in binding to the substrate (Additional file 1: Fig. S3B). In addition, both *Ss*Chi18A and *Ss*Chi18C have open and deep active binding clefts, while *Ss*Chi18B has a shallow substrate-binding cleft (Fig. 4b). On the other hand, the results of sequence alignment also showed that the active center of *Ss*Chi18C is quite distinct from those of *Ss*Chi18A and *Ss*Chi18B, where *Ss*Chi18C only has a catalytic motif of DXDXE instead of the reported conserved motif DXXDXDXE (Additional file 1: Fig. S4).

In the close-up view of the active site, the number of interactions for *Ss*Chi18A, *Ss*Chi18B, and *Ss*Chi18C with different subsites are different, which are summarized in Additional file 1: Table S2, with significant sub-family specificity (Additional file 1: Fig. S5). For *Ss*Chi18A, the interactions are distributed on subsites − 6 to + 2, forming a total of 27 interactions, but mainly concentrated on subsites + 1 to + 2, with a loss of overall substrate-binding capacity on negative subsites. The numbers of interactions for *Ss*Chi18B are greater than those with *Ss*Chi18A, forming a total of 30 interactions on subsites − 5 to + 2, with most at subsites − 2 and − 1. For *Ss*Chi18C, there are 24 interactions on subsites − 3 to + 2, with most at subsites − 3 and − 2. This result can explain why *Ss*Chi18B can degrade G3, while *Ss*Chi18C mainly degrades G4, as the binding strength of subsite − 1 directly affects the binding ability of the enzyme to the oligosaccharide substrate [28, 29]. Altogether, our data showed that the catalytic domains of *Ss*Chi18A, *Ss*Chi18B, and *Ss*Chi18C indeed have different active architectures, which could directly affect the binding capacities and catalytic activities.

### Chitinases from different GH18 subfamilies functioned synergistically

**Fig. 5:**
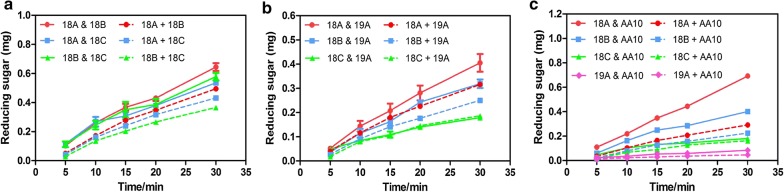
Synergy experiments of different chitinases. The reaction mixture (200 μL total volume) contained 100 μL of 2 mg/mL colloidal chitin, supplemented with 50 μL enzyme solution and/or, in control reactions, 50 μL of the reaction buffer, pH 5.0. The final concentration of each enzyme was 2.5 nmol/mL. The reaction mixtures were incubated at 60 °C for 30 min. The experiments labeled with “&” (solid lines) represent the amount of reducing sugar produced when the two enzymes act on the substrate together. The curves labeled with “+” (dashed lines) represent product levels calculated by adding product formation by each of the individual enzymes. 18A indicates *Ss*Chi18A, 18B indicates *Ss*Chi18B, 18C indicates *Ss*Chi18C, 19A indicates *Ss*Chi19A, and AA10 indicates *Ss*LPMO10A

The observations that chitinases from different GH18 subfamilies exhibited different preferences during the degradation of specific substrates and different active structures, implied they might play different roles in the chitin-degrading process to increase efficiency. To verify this, synergy experiments were performed. Among the GH18 family chitinases, the synergic action between *Ss*Chi18B and *S*sChi18C was more obvious than that between *S*sChi18A and *S*sChi18B, or *S*sChi18A and *S*sChi18C (Fig. 5a). When mixed with *Ss*Chi19A, *Ss*Chi18C did not have any cooperation with it, while *Ss*Chi18A and *Ss*Chi18B showed some increase in the total activity (Fig. 5b). In addition, *Ss*LPMO10A also had a promoting effect on the actions of *S*sChi18A and *S*sChi18B, whereas no effect was observed on *S*sChi18C and *S*sChi19A (Fig. 5c). Taken together, our data indicated that *S*sChi18A plays a more important role in the chitinolytic machinery of *Streptomyces* sp. F-3, which is likely essential for the effective degradation of natural chitin.

## Discussion

Although seven genes are predicted to code for secreted enzymes involved in chitin degradation, only six chitin degradation-related proteins were identified by proteomics, indicating lower expression or no function for the missing *Ss*LPMO10B when chitin was used as the sole carbon source. Among these detected chitin degradation-related enzymes, the GH18 chitin hydrolases are widely present in various chitin-degrading microorganisms and play an important role in the degradation of chitin substrates [12, 30]. Different from *S. marcescens* and *C. japonicus*, the GH18 chitinases secreted by *Streptomyces* sp. F-3 have some special accessory-binding domains. For example, both *Ss*Chi18A and *Ss*Chi18B have a FN3 (PF00041) domain, which universally exists in the chitinases from terrestrial bacteria of the phyla Actinomycetes and Firmicutes, and the function of this domain may be related to the interactions between terrestrial bacterial chitinases and fungal chitin resources [10]. However, another FN3 (PF08329) domain found in the GH18 family chitin hydrolase ChiA of *S. marcescens* contains a variety of aromatic amino acids, which could enhance chitin hydrolysis [31]. Besides FN3, the CBM domains also widely occur in the GH18 family chitinases, which display versatile functions [32, 33]. For example, the CBM2 domain found in both *Ss*Chi18A and *Ss*Chi18C is reported to increase the binding ability of the enzymes to chitin [33]. Consistent with this, our biochemical data for *Ss*Chi18A and *Ss*Chi18C showed higher activity on chitin substrates than *Ss*Chi18A_cat_ and *Ss*Chi18C_cat_ (data not shown). Therefore, this particular combination of domains in *Streptomyces* sp. F-3 may result in more efficient degradation of chitin in nature.

The catalytic domains of the GH18 chitinases in *Streptomyces* sp. F-3 are classified into three subfamilies (*Ss*Chi18A, *Ss*Chi18B, and *Ss*Chi18C), which are also found in both *S. marcescens* and *C. japonicus*. Based on structural and biochemical data, *Sm*ChiA and *Sm*ChiB of *S. marcescens* and *Cj*Chi18D of *C. japonicus* are thought to be processive, and it has been shown that such processivity is essential for the efficient degradation of crystalline chitin [12, 34]. Similarly, the *Ss*Chi18A also seems to be a processive chitinase, which can be concluded from the following results. (i) *Ss*Chi18A shows a close relationship to the processive chitinase *Cj*Chi18D based on phylogenetic analysis; (ii) the catalytic domain of *Ss*Chi18A has a large α + β domain (about 81 amino acids) insertion at loop 7 and an open deep active binding cleft, which are specific hallmarks of processive enzymes [25, 35], and its active architecture is distributed with similar aromatic amino acids with the processive chitinase *Sm*ChiA from *S. marcescens* and *Cj*Chi18D from *C. japonicus* [12, 24]; (iii) the conserved motif SXGGW occurs in *Ss*Chi18A_cat_, and the Trp residue has been demonstrated essential for processivity [26, 27]; (iv) FACE analysis showed that *Ss*Chi18A can processively hydrolyze colloidal chitin and CHOSs to chitobiose. *Ss*Chi18B has a special β hairpin subdomain at loop 6 and a relatively shallow active site binding cleft, in which its aromatic amino acids are concentrated in the vicinity of the catalytic center. This resembles the predicted structure of *Sm*ChiC from *S. marcescens* [27]. Different from *Ss*Chi18A and *Ss*Chi18B which have eight loops, *Ss*Chi18C only has seven loops without the α + β domain at loop 7. Alternatively, it has a long chain in loop 7, which may compensate for the function of loop 8 in *Ss*Chi18A and *Ss*Chi18B. Trp-257 and Arg-261 in loop 7 were found to increase the binding ability to subsites − 1 and − 2. Interestingly, in *Ss*Chi18C, the first conserved aspartate in the catalytic DXXDXDXE motif is replaced by a threonine. Since *Ss*Chi18C is an active enzyme, this first aspartate is apparently not essential for its activity.

As mentioned above, the three GH18 family chitinases revealed clear functional and structural differences, and as expected, these GH18 enzymes of *Streptomyces* sp. F-3 act synergistically during the degradation of chitin. This synergy was also observed in *S. marcescens* and *C. japonicus*, which is likely due to the collaboration between *endo*- and *exo*-acting enzymes [36]. Although the *endo*/*exo* characteristics of the *Streptomyces* enzymes were not addressed in this study, the domain structures and active site architectures of *Ss*GH18s suggest an *endo*/*exo* complementarity that is similar to that observed in *S. marcescens* and *C. japonicus*. Similar to *Sm*LPMO10A of *S. marcescens* which promotes the hydrolysis of crystalline chitin by *Sm*ChiA and *Sm*ChiC [7], *Ss*LPMO10A secreted by *Streptomyces* sp. F-3 also showed a significant synergy with *Ss*Chi18A and *Ss*Chi18B, suggesting a key function of *Ss*LPMO10A for the bioconversion of chitin.

## Conclusions

Overall, in this study, the substrate-binding and catalytic patterns and the synergistic structures of different chitinases in *Streptomyces* sp. F-3 were analyzed to reveal the biological basis of the synergy of enzymes. The results showed that *Ss*Chi18A, *Ss*Chi18B, and *Ss*Chi18C belong to three different GH18 subfamilies, with different biological structures and specific substrate-binding modes. Among them, *Ss*Chi18A may act processively and function primarily during chitin degradation, and synergy with the proposed non-processive *Ss*Chi18B and *Ss*Chi18C. In addition, *Ss*LPMO10A of the enzyme system also plays an important role in the high efficiency of chitin hydrolysis. Therefore, the complete study of these thermophilic chitin-degrading enzymes will lay a theoretical foundation for the efficient industrialized transformation of natural chitin.

## Materials and methods

### Strain and medium

*Streptomyces* sp. F-3, a thermophilic chitinase producer, was isolated from an alkaline-composting environment from Yucheng, Shandong, China [19]. This strain was cultured at 50 °C in Luria–Bertani medium (10 g of tryptone, 5 g of yeast extract, and 5 g of NaCl in 1 L of deionized water, pH 7.0).

### Substrate materials

Chitin and chitosan were purchased from Sigma-Aldrich Corporation (St. Louis, MO, USA). Chitin oligosaccharides (*N*-acetylglucosamine, GlcNAc2, GlcNAc3, GlcNAc4, GlcNAc5, and GlcNAc6) were purchased from Qingdao Bz Oligo Biotech Co., Ltd. (Qingdao, China). Colloidal chitin was produced as described by Hirano and Nagao [37].

### Analysis of the secretomes by liquid chromatography tandem-mass spectrometry

The strain was grown for 60 h in liquid culture with shaking (2 g yeast extract, 5 g tryptone, 3 g NaNO_3_, 1.0 g K_2_HPO_4_, 0.5 g MgSO_4_·7H_2_O, 0.5 g KCl, 0.01 g FeSO_4_·7H_2_O, in 1 L of distilled water, pH = 7.5) using 1% colloidal chitin as the sole carbon source. All cultures were incubated at 50 °C with an aeration of 200 rpm, and then the secreted proteins at 48 h were analyzed by liquid chromatography tandem-mass spectrometry using a method described elsewhere [38]. A label-free quantification method was used and the relative abundance of proteins was characterized by peptide spectrum matches (PSMs). According to earlier studies, there was a linear correlation between peptide spectrum matches and protein abundance [39]. On this basis, the relative quantitative analysis of proteins was achieved by calculating the ratio of the PSM value of a single protein to the total PSM value of the extracellularly detected proteins.

### Gene cloning, expression, and protein purification

Total DNA from the mycelia of *Streptomyces* sp. F-3 was extracted using the E.Z.N.A.^®^ Bacterial DNA Kit (Omega Bio-Tek, Inc., Norcross, GA, USA). Primers were designed based on genes of chitinase exclude of signal peptides. *Escherichia coli* strains DH5α and BL21 (DE3) were used as transformation and expression hosts, respectively, while the *Ss*LPMO10A was expressed and purified as previously described by Forsberg et al. to ensure that the first amino acid at the N-terminus of the protein is histidine [40]. The plasmid pET28A was used as a protein cloning and expression vector. Kanamycin, when added, was used at a final concentration of 50 μg/mL. *E. coli* strain BL21 (DE3) harboring recombinant plasmids was cultivated in Luria–Bertani medium containing 50 μg/mL of kanamycin for 3 h at 37 °C until the optical density at 600 nm reached approximately 0.6, and then induced for over-expression with 0.1 mM isopropyl-β-d-thiogalactopyranoside (IPTG) and incubated for 20 h at 20 °C. Cells were harvested by centrifugation (8000×*g* for 10 min at 4 °C), washed with phosphate-buffered saline (0.3 M NaCl, 50 mM NaH_2_PO_4_, with sufficient sodium hydroxide to adjust the pH to 8.0), and then resuspended in the same buffer. The cells were disrupted by sonication and the supernatant was obtained by centrifugation (11,000×*g* for 1 h at 4 °C). The supernatant was loaded onto a Co^2+^ TALON^®^ Metal Affinity Resin (TaKaRa Biotechnology (Dalian) Co., Ltd., Dalian, China). The column was washed with 100 mL of phosphate-buffered saline (pH 8.0) containing 5 mM and 10 mM imidazole, respectively. Co^2+^ TALON-bound enzyme was eluted with 20 mM imidazole in the same buffer. The eluted fractions were then centrifugally dialyzed using a 3 K Macrosep Advance ultrafiltration centrifuge tube (Pall Corporation, Port Washington, NY, USA) to remove imidazole. The enzyme was stored at − 20 °C until used. Sodium dodecyl sulfate–polyacrylamide gel electrophoresis (SDS–PAGE) was carried out to determine the purity of the enzyme solution.

### Chitinase activity assay

Chitinase activity was measured by detecting the amount of reducing sugar released from colloidal chitin as substrate [41]. Unless otherwise stated, the reaction mixture containing 100 μL of 10 mg/mL colloidal chitin, which was dissolved in 50 mM Na_2_HPO_4_-citric acid buffer at pH 5.0, and 100 μL of suitably diluted enzyme solution at a final volume of 200 μL was incubated at 60 °C for 30 min. The reaction was stopped by the addition of 300 μL of modified dinitrosalicylic (DNS) acid, and then the mixture was boiled for 10 min, chilled, and centrifuged to remove insoluble chitin. The amount of reducing sugar in the supernatant was determined using the modified dinitrosalicylic acid method [42]. With the use of CHOSs as substrates, the reaction mixture containing 90 μL of 1 mg/mL CHOS and suitably diluted enzyme solution (10 μL) at a final volume of 100 μL was incubated at 60 °C for 30 min. One unit of enzymatic activity (*U*) was defined as the amount of enzyme required to produce 1 μmol of reducing sugar from colloidal chitin per min under the specified assay conditions. All reactions were performed in triplicates. In reactions containing *Ss*LPMO10A, ascorbic acid was added to a final concentration of 1.0 mM (external electron donor).

### Analysis of hydrolysis products

The reaction products for various chitin oligosaccharides and colloidal chitin were analyzed by fluorophore-assisted carbohydrate electrophoresis (FACE). First, the reaction hydrolysate or CHOS (5 μL) was treated with 7-amino-1,3-naphthalenedisulfonic acid monopotassium salt monohydrate in 15% acetic acid (0.2 M, 5 μL) and reacted in the dark for more than 1 h. NaCNBH_3_ solution (1 M, 5 μL) was added to the mixture, which was then incubated at 42 °C overnight. Labeled products (7 μL/well) were loaded for electrophoresis. The ChemiDocTM MP imaging system (Bio-Rad Laboratories, Hercules, CA, USA) was used to scan the images, which were stored in tagged image format files.

### Sequence analysis

All protein sequences used in the present study were obtained from the UniProt database (https://www.uniprot.org/). Multiple sequence alignments were performed using the Clustal Omega multiple sequence alignment algorithm (http://www.clustal.org/omega/). The phylogenetic tree was constructed using Molecular Evolutionary Genetics Analysis software [43] and optimized with the Interactive Tree of Life online tool (http://itol.embl.de/).

### Structural bioinformatics analysis

The structures of chitin degradation-related proteins were constructed using the SWISS-MODEL structural bioinformatics web-server (https://swissmodel.expasy.org/) and displayed with the PyMOL molecular visualization system (http://www.pymol.org). Simulations of molecular dynamics (MD) were performed using the GROMACS software package (http://www.gromacs.org/). The biophysical properties were analyzed using the internal tools in GROMACS. After removing the overall translational and rotational motions by superimposing the Cα atoms of each snapshot structure onto the starting structure using least-squares fitting, the root mean square deviation (RMSD; g_rms) were calculated to analyze structural stability. Lastly, 50-ns molecular dynamic simulations were performed to further equilibrate the systems. The selected protein structures at 50 ns require substrate docking in PyMOL. The *Ss*Chi18A–substrate complex was constructed by docking the chitooctaose of *Sm*ChiA (PDB: 1EHN) into the active site cleft, and “docking” means putting the ligand which was obtained from the crystal structure of enzyme-ligand substrate complexes into the structural models. The *Ss*Chi18B–substrate complex was constructed by docking the chitoheptaose of *Mm*Chi60 (PDB: 4MB4) and hevamine A (PDB: 1KQY) into the active site cleft. The *Ss*Chi18C–substrate complex was constructed by docking the chitopentaose of *Pf*ChiA (PDB: 3A4W) into the active site cleft. The *Ss*Chi19A–substrate complex was constructed by docking the chitotetraose of *Gc*ChiA (PDB: 3WH1) into the active site cleft. Based on a cut-off of 5 Å, amino acid residues having interactions with the substrate were selected for further analysis. Then, with the same method, the sequence profiles of the active site architectures of the three GH18 subfamilies were created using the web-based application WebLogo (http://weblogo.berkeley.edu/).

## Additional file

**Additional file 1.** Additional figures and tables.

## Data Availability

All data generated or analyzed during this study are included in this published article.

## Abbreviations

CHOS: chitin oligosaccharide
LPMO: lytic polysaccharide monooxygenases
GH: glycoside hydrolase
AA: auxiliary activities
CBM: carbohydrate-binding module
FN3: fibronectin type III domain
SubA: sub-family A
GlcNAc (NAG): *N*-acetyl-β-d-glucosamine
RMSD: root mean square deviation
FACE: fluorescence-assisted carbohydrate electrophoresis
