# Supplementary material for: A novel thermostable chitinolytic machinery of Streptomyces sp. F-3 consisting of chitinases with different action modes
Source: Biotechnol Biofuels. 2019 Jun 3;12:136. doi: 10.1186/s13068-019-1472-1 (PMC6545677; doi:10.1186/s13068-019-1472-1)
Supplement: Supplementary file 1 — Additional file 1. Additional figures and tables. [file 13068_2019_1472_MOESM1_ESM.docx]

**Additional files**

## A novel thermostable chitinolytic machinery of *Streptomyces* sp. F-3 consisting of chitinases with different action modes

Xiaomeng Sun^1^, Yingjie Li^1^, Zhennan Tian^1^, Yuanchao Qian^1^, Huaiqiang Zhang^1*^, Lushan Wang^1*^

^1^*State Key Laboratory of Microbial Technology, Microbial Technology Institute, Shandong University, No. 72 Jimo Binhai Road, Qingdao 266237, Shandong, People’s Republic of China*

*Corresponding author: Huaiqiang Zhang; Lushan Wang

The list of e-mails of the authors:

Xiaomeng Sun: 18766163892@163.com

Yingjie Li: sdliyingjie@163.com

Zhennan Tian: zhennant@163.com

Yuanchao Qian: qianyuanchao@hotmail.com

Huaiqiang Zhang: zhq@sdu.edu.cn

Lushan Wang: lswang@sdu.edu.cn


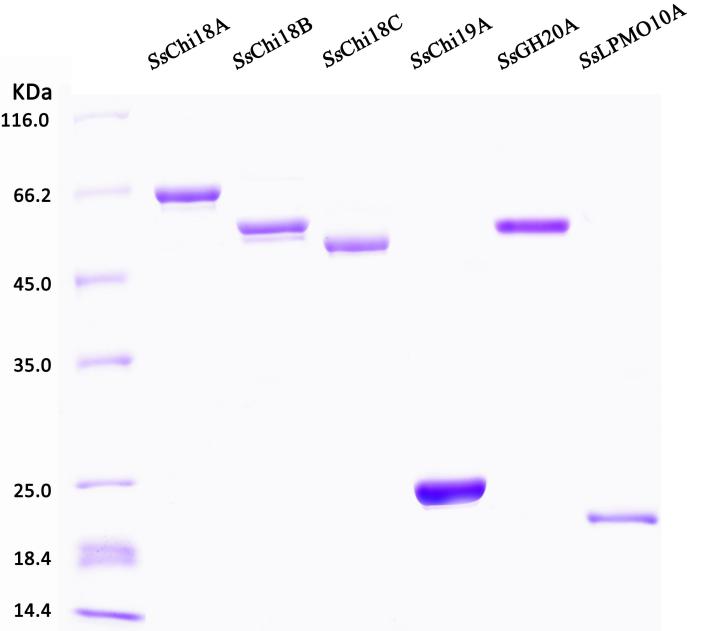


**Fig. S1 SDS-PAGE analysis of six chitin-degradation related enzymes from *Streptomyces* sp. F-3 expressed in *E. coli* strain BL21(DE3).**


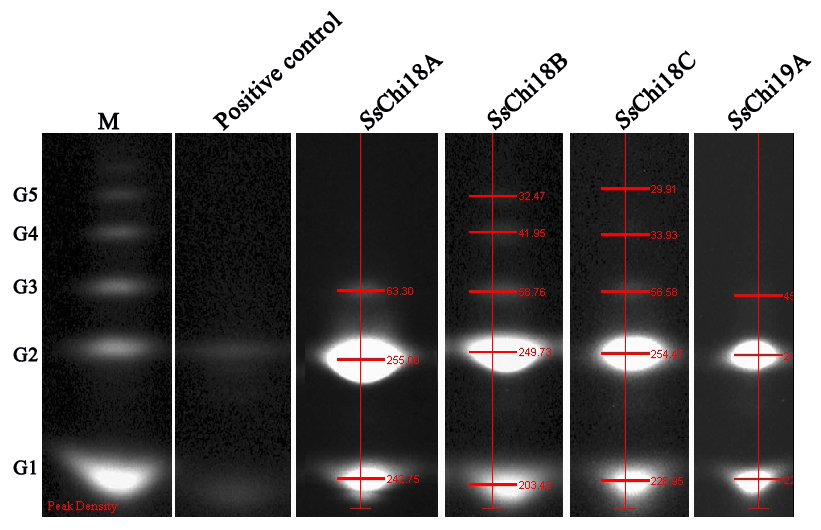


**Fig. S2 FACE analysis of hydrolysis products by chitinases from colloidal chitin.** The reaction mixture (200 μL) containing 100 μL of 1 mg/mL substrate and purified enzyme (100 μL, 0.01 μmol/mL) for 20 min at 60°C.


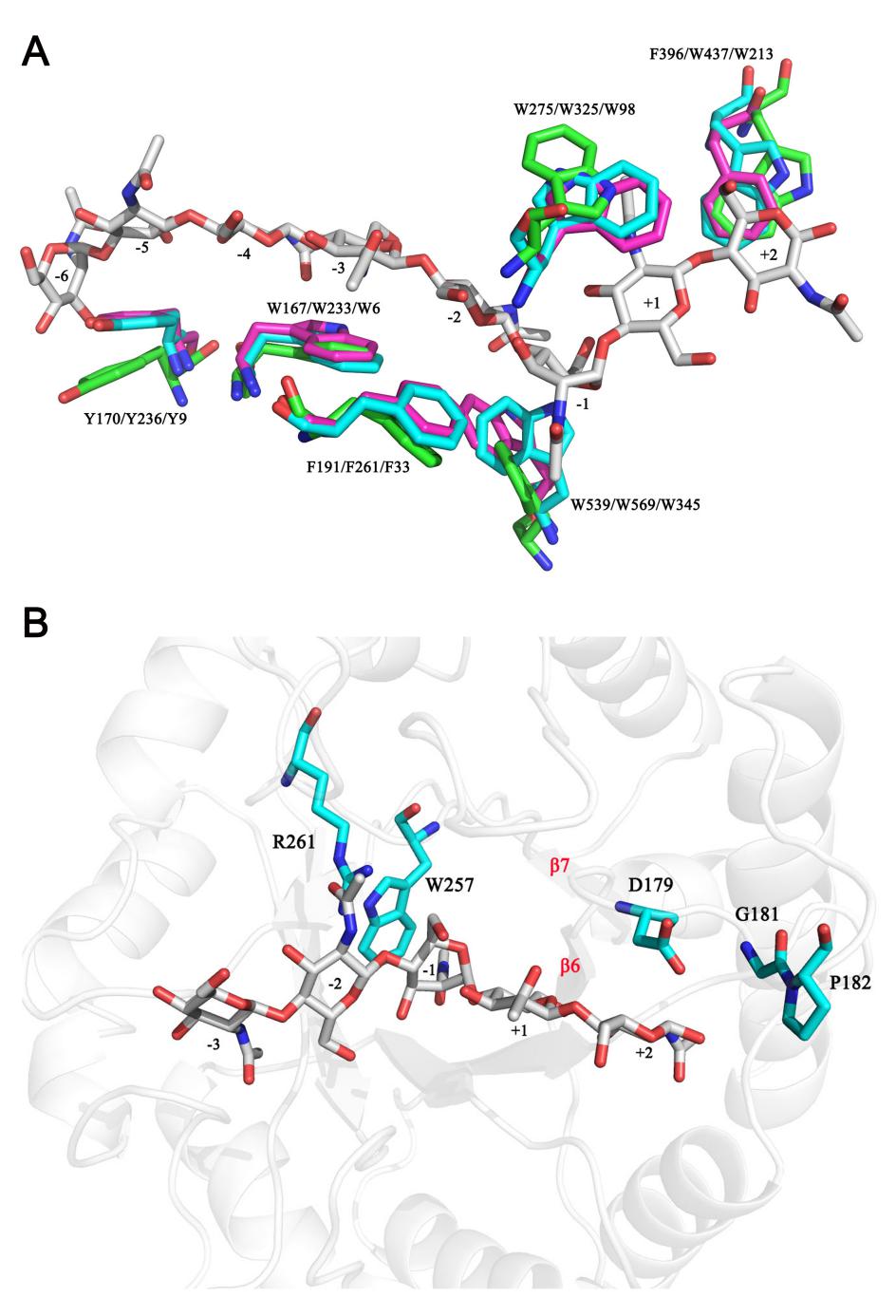


**Fig. S3 Structural bioinformatics analysis of the GH18 chitinases.** (A) Aromatic amino acids lining the substrate-binding cleft of *Sm*ChiA (magenta sticks), *Cj*ChiD (cyan sticks) and *Ss*Chi18A (green sticks), number labels in the same order. The substrate (GlcNAc)8 bound to the structure is shown in gray stick representation, and subsites which the sugars are bound to are indicated. Nitrogen and oxygen atoms are colored blue and red, respectively. Notably, the aromatic amino acids in the +2 subsites of the *Sm*ChiA is Phe-396, while in *Cj*ChiD and *Ss*Chi18A are replaced with Trp-437 and Trp-213. (B) Amino acids that interacts with the substrates lining the substrate-binding cleft of *Ss*Chi18C (cyan sticks). Asp-179, Gly-181, and Pro-182 were located on loop 6, which acted with the subsites +1 and +2; Trp-257 and Arg-261 were located on loop 7, which acted with the subsites -1 and -2. The substrate (GlcNAc)5 bound to the structure is shown in gray stick representation. Nitrogen and oxygen atoms are colored blue and red, respectively.


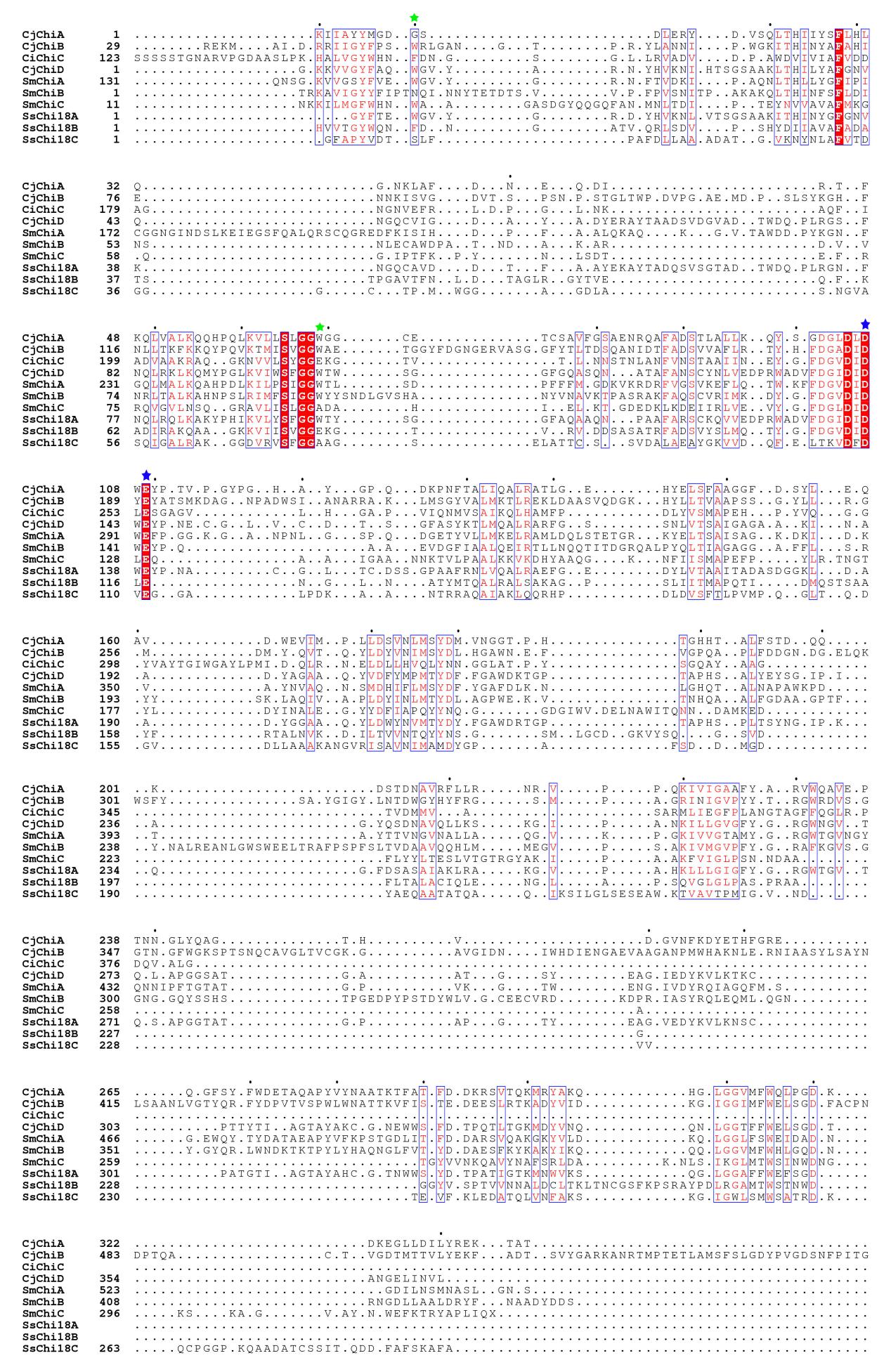


**Fig. S4 Sequence alignment.** The catalytic domains of three GH18 family chitinases from *Streptomyces* sp. F-3 are aligned with the catalytic domains of well-characterized chitinases from *S. marcescens* and *C. japonicus*. Fully conserved residues are shown in red background, and blue star indicate the catalytic Asp and Glu acting as the catalytic acid/base. Trp residues in *Sm*ChiA and *Cj*ChiD are known to be essential for processivity and shown with green stars.


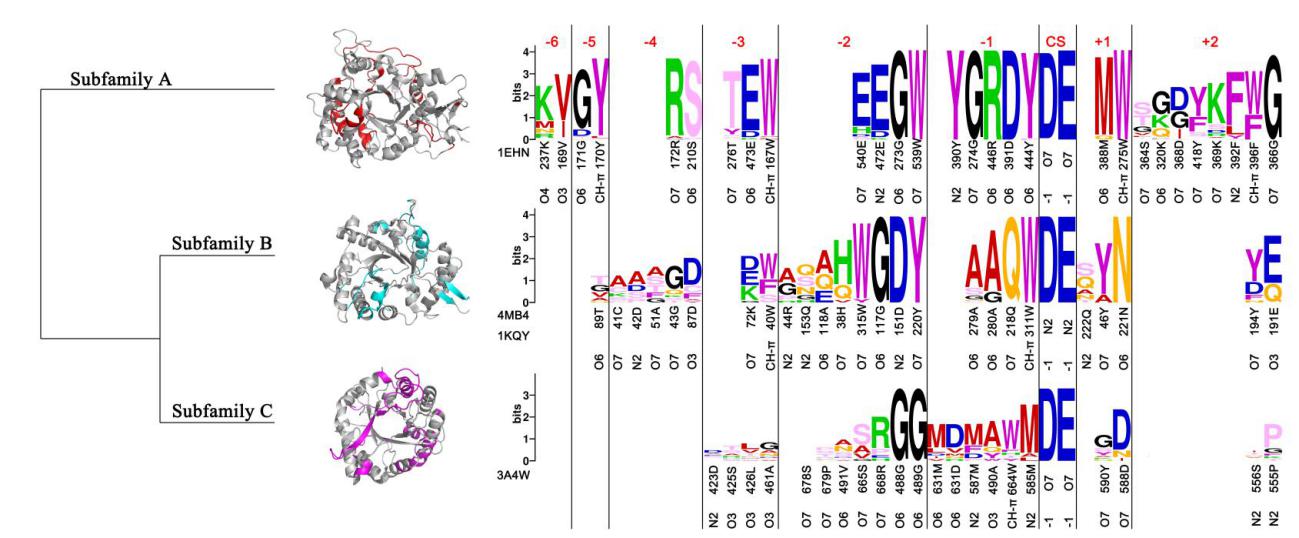


**Fig. S5 Sequence logos of the active sites of GH18 subfamilies A, B, and C.** The model proteins for the alignment include *Sm*ChiA (PDB: 1EHN) of *S. marcescens*, *Mm*Chi60 (PDB: 4MB4) of *Moritella marina* (docked with the ligand from PDB: 1KQY), and *Pf*ChiA (PDB: 3A4W) of *Pyrococcus furiosus* DSM 3638. The PDB IDs are marked on the left bottom of each panel. For each model protein, residues located within 5 Å of certain substrate subsites are identified as having interactions with the certain subsite and residues are displayed under the subsite with which they interact. The collection of all residues makes up the active site. The sequence number of the residues in each model protein as well as the substrate atoms with which they interact are listed below each logo.

**Table S1 Chitin-degradation related enzymes detected by mass spectrometry**

| Enzymes | Uniprot ID | Signal | # AAs | MW [kDa] | *S. marcescens*  (e-value, identity) | *C. japonicus*  (e-value, identity) | ^a^PSMs (%) |
| --- | --- | --- | --- | --- | --- | --- | --- |
| *Ss*Chi18A | A0A171ASV3 | Yes | 607 | 63.89 | *Sm*ChiA  (7e-48, 29.7%) | *Cj*ChiD  (0.0, 67.81%) | 2.10 |
| *Ss*Chi18B | A0A170ZR08 | Yes | 569 | 58.51 | *Sm*ChiC  (2e-25, 26.07%) | *Cj*ChiC  (5e-69, 39.39%) | 0.82 |
| *Ss*Chi18C | A0A170UCS3 | Yes | 486 | 49.95 | *Sm*ChiC  (7e-06, 22.60%) | *Cj*ChiC  (2e-08, 25.56%) | 0.01 |
| *Ss*Chi19A | A0A170ZXU8 | Yes | 231 | 24.74 | *Sm*Chi19A  (2e-06, 26.67%) | *Cj*Chi19A  (4e-81, 58.06%) | 0.40 |
| *Ss*GH20A | A0A170UF08 | Yes | 563 | 61.64 | *Sm*GH20A  (1e-45, 27.58%) | *Cj*GH20B  (2e-48, 28.36%) | 1.17 |
| *Ss*GH20B | A0A170U6E0 | No | 546 | 60.26 | *Sm*GH20B  (7e-47, 28.39%) | *Cj*GH20A  (4e-61, 37.05%) | ^b^ND |
| *Ss*GH20C | A0A170XSD6 | No | 461 | 50.5 | *Sm*GH20A  (1e-18, 23.44%) | *Cj*GH20A  (1e-27, 33.81%) | ND |
| *Ss*LPMO10A | A0A170UBE6 | Yes | 201 | 21.46 | *Sm*LPMO10B  (8e-50, 44.57%) | *Sm*LPMO10A  (6e-19, 30.24%) | 0.65 |
| *Ss*LPMO10B | A0A171AT58 | Yes | 359 | 36.2 | *Sm*LPMO10B  (5e-17, 29.47%)- | *Sm*LPMO10A  (6e-50, 45.93%) | ND |

^a^PSMs (peptide-spectrum matching), is the matching of the spectrum and the peptide, which can be used to indicate the content of extracellular proteins.

^b^ND indicated not detectable.

**Table S2 Residues interacting with substrate subsites**

| Subsites | Atoms | ^a^Residues having interactions with subsites | | | ^b^Number of interactions |
| --- | --- | --- | --- | --- | --- |
|  |  | *Ss*Chi18A | *Ss*Chi18B | *Ss*Chi18C |  |
| -6 | O4 | 53K |  |  | 1, 0, 0 |
| -5 | O6 |  | 48D, 50A, 51G |  | 1, 3, 0 |
|  | CH-π | 9Y |  |  |  |
| -4 | N2 | 11R |  |  | 2, 5, 0 |
|  | O3 |  | 48D |  |  |
|  | O6 |  | 82K |  |  |
|  | O7 | 11R | 9N, 11D, 51G |  |  |
| -3 | N2 |  | 82K | 9T, 10S, 45G | 2, 4, 9 |
|  | O3 |  | 82K | 45G, 46G, 47A |  |
|  | O4 |  |  | 10S, 11L, 46G |  |
|  | O6 | 11R, 286Y |  |  |  |
|  | O7 |  | 81E | 45G |  |
|  | CH-π |  | 10F |  |  |
| -2 | N2 |  |  | 8D, 261R | 2, 6, 7 |
|  | O3 | 97W |  |  |  |
|  | O6 |  | 80G, 81E | 76A, 77A |  |
|  | O7 | 346E | 8Q, 12N, 225A, 271W | 8D, 261R, 275A |  |
| -1 | N2 |  | 175Q | 148M | 5, 7, 5 |
|  | O3 |  | 145A |  |  |
|  | O6 | 208D, 263R, 287E | 225A, 226A | 176M, 223M, 227N |  |
|  | O7 | 176A | 145A, 177Y |  |  |
|  | CH-π | 345W | 267W | 257W |  |
| +1 | N2 |  |  |  | 7, 3, 2 |
|  | O3 |  |  |  |  |
|  | O6 | 178T, 179A, 205M, 207Y | 175Q, 177Y, 178N | 147P, 148M |  |
|  | O7 | 208D, 263R, 287E |  |  |  |
| +2 | N2 | 186K, 207Y, 208D, 209Y | 147Q | 179D | 7, 2, 1 |
|  | O3 | 178T, 186K | 147Q |  |  |
|  | O6 |  |  |  |  |
|  | O7 | 180D |  |  |  |

^a^In terms of a certain enzyme, residues present within 5 Å of each substrate subsite were identified as having interactions with the appointed subsite.

^b^The numbers "x, y, z" represent the number of interactions; "x" for *Ss*Chi18A, "y" for *Ss*Chi18B, "z" for *Ss*Chi18C.
